# Adsorption and detoxification of poly- and perfluoroalkyl substances (PFAS) with hydrophobic bentonite clays

**DOI:** 10.1007/s11356-026-37545-x

**Published:** 2026-02-28

**Authors:** Johnson Olaleye Oladele, Timothy D. Phillips

**Affiliations:** 1 Department of Veterinary Physiology and Pharmacology, College of Veterinary Medicine and Biomedical Sciences, Texas A&M University, College Station, TX 77843 USA; 2 Interdisciplinary Faculty of Toxicology, College of Veterinary Medicine and Biomedical Sciences, Texas A&M University, College Station, TX 77843 USA

**Keywords:** PFAS, Adsorption, Organoclays, Ecotoxicity

## Abstract

PFAS is a class of emerging contaminants, and their exposure has been linked to many deleterious health effects which pose major threats to public health. Dietary intake has been recognized as the principal source of PFAS exposure to animals and humans while drinking water is the major route. Given the persistent nature of PFAS in water and the environment, this study was designed to provide effective and potentially scalable strategies to mitigate PFAS toxicity using organoclays. The study has been designed to investigate and characterize the adsorption behavior of four PFAS (PFOA, PFOS, PFBS, and GenX) onto active binding surfaces of four commercially available organoclay products called ENVIRO-TROL™ (ENT), ORGANO-TROL™ (OT2), and AGRI-TROL™ (ATB and ATM). This study evaluates key PFAS binding markers for these organoclays including Gibbs free energy (∆G), adsorption capacity, and binding affinity for PFAS. These parameters served as indicators for predicting the bioavailability of PFAS under the experimental settings. Additionally, the proof-of-concept for the protective role of the organoclay in PFAS-mediated toxicity was evaluated using living organisms (*Hydra vulgaris* and *Lemna minor*) as sensitive ecotoxicology models. The results showed varied adsorption behavior from pH 2 to 10. All the organoclays demonstrated the highest PFAS binding at pH 2. OT2 showed the highest adsorption for PFOA (78%), PFOS (47%), GenX (54%), and PFBS (66%), followed by ATM, while ATB and ENT showed slightly lower binding. Isotherm data indicated that all the binding followed a Freundlich model with high adsorption capacity. Moreover, they were all thermodynamically favorable with ∆G values ranging from − /20.00/ to − /30.96/kJ/mol. In vivo data indicated severe toxicity of PFAS exposure to *hydra vulgaris* and *Lemna minor*; however, inclusion of the organoclays into the aqueous media of hydra and lemna resulted in over 90 to 100% protection from PFAS toxicity. This finding implies that these organoclays may be used in environmental remediation applications to adsorb PFAS from polluted water and bind and detain it in contaminated soil.

**Graphical Abstract:**

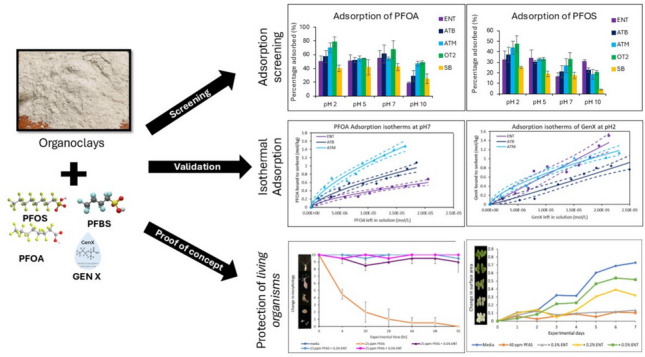

**Supplementary Information:**

The online version contains supplementary material available at 10.1007/s11356-026-37545-x.

## Introduction

Poly- and perfluoroalkyl substances (PFAS) can penetrate the food chain, soil and plants via exposure to contaminated soil, drinking water, groundwater, wastewater, and industrial effluents (Mukhopadhyay et al. 2021). These substances represent an extensive class of more than 5000 thermally stable compounds recognized for their persistence in the environment and globally dispersed as persistent organic pollutants (POPs), with many applications across various industrial fields (Buck et al. 2011; Liu et al. 2019). PFAS are commonly found in products designed to resist water, dirt, and grease, including firefighting foam, personal care products, cosmetics, food packaging materials, and fabrics (Bolan et al. 2021). The levels of PFAS contamination vary significantly across different water environments such as drinking water, groundwater, stormwater, seas, rivers, and lakes (Kabore et al. 2018). Due to their extensive use and unintentional discharge, PFAS continue to be a major contributor to groundwater and soil pollution (EPA 2021).

PFAS molecules exhibit a range of carbon chain lengths, and in the case of poly- and perfluoroalkyl acids, hydrogen atoms along the backbone are substituted with fluorine atoms (Buck et al. 2011). Importantly, these compounds also have ionizable functional groups such as sulfonic and carboxylic acid moieties. Based on the number of carbon atoms in their molecular structure, PFAS are generally classified as short-chain (≤ 5 carbon atoms) and long-chain (≥ 6 carbon atoms) (OECD/UNEP 2013). Short-chain PFAS tend to be more water-soluble and mobile in aqueous environments compared to their long-chain counterparts, thus making their elimination from the environment very difficult (Gagliano, et al. 2020). Upon their release into the environment, these substances can migrate through water and soil where they are taken up by animals (Navarro et al. 2016; Zhang et al. 2019), and plants (Costello and Lee 2020). Consequently, this uptake results in their bioaccumulation and biomagnification through the food web, which often reach high concentrations in top predators and humans (Garg et al. 2020; Liew et al. 2018; Sunderland et al. 2019). Long-chain PFAS, particularly PFOS with 8 carbon atoms, demonstrate a greater potential for biomagnification than short-chain PFAS. However, due to higher solubility, short-chain PFAS are more widely dispersed in environmental matrices (Sunderland et al. 2019; Wang et al. 2015).

Human and animal exposure to PFAS (with biological half-lives of approximately 2.7–5.3 years) occurs primarily through ingestion of contaminated water and drinking water (Li et al. 2018; Domingo and Nadal 2019). Important routes to this exposure include consumption of contaminated seafood, and fish as well as agricultural products like vegetables, soy, corn, and wheat, along with PFAS-polluted potable water supplies (Christensen et al. 2017; Ghisi et al. 2019). Drinking water is often the main pathway of PFAS exposure to local populations that have proximity to contamination sites. This is largely due to the limited capacity of the conventional wastewater treatment systems to effectively eliminate these substances (Arvaniti and Stasinakis 2015; Sunderland et al. 2019). The removal of PFAS, regardless of their chain length, is essential given their ability to bioaccumulate and cause deleterious health effects in animals and humans.

Epidemiological studies have linked specific PFAS compounds such as perfluorooctane sulfonate (PFOS) and perfluorooctanoic acid (PFOA) to disease conditions such as cancer, hypertension, high cholesterol, and thyroid problems in humans (Crawford et al. 2017). Numerous remediation technologies have been investigated to clean up PFAS-contaminated water including photo-reductive destruction under UV irradiation (Chen et al. 2020), reverse osmosis, adsorption (Sorengård et al. 2020), biological degradation (aerobic and anaerobic), and advanced oxidation processes. However, these methods often face substantial setbacks stemming from intensive energy requirement, high operational cost, and difficulty for field scale application (Lee et al. 2012). Among the available techniques, adsorption has emerged as a promising and cost-effective approach, owing to its efficiency and selectivity in removing PFAS from water at large-scale field applications (Schroder et al. 2010).

Clay minerals have garnered significant attention in environmental remediation research due to their natural abundance throughout continents, eco-friendliness, and cost-effectiveness (Sarkar et al. 2019; Oladele et al. 2024a, b; Oladele et al. 2025a). Despite these merits, unmodified clay minerals often demonstrate limited efficiency in removal of bulky contaminants from aqueous systems. Thus, to enhance their adsorption capacity, clays are often functionalized by either inorganic or organic materials, thereby improving their surface properties and functionality, selectivity, and overall adsorption performance (Sarkar et al. 2019; Wang et al. 2021; Xenophontos et al. 2025; Oladele et al. 2024a; Oladele et al. 2025a, b). This study investigates four organoclays as PFAS binding clay-products containing different levels of a quaternary ammonium amine. The capacity of the clay-based products to bind four common PFAS including PFOA (CF_3_(CF_2_)_6_COOH), PFOS (C_8_F_17_O_3_S), PFBS (perfluorobutanesulfonic acid) (C_4_HF_9_O_3_S), and GenX (hexafluoropropylene oxide dimer acid or HFPO-DA) (C_6_HF_11_O_3_) were investigated. PFOA and PFOS are long chain PFAS with eight carbon atoms each while PFBS and GenX have four and six carbon atoms respectively in their structures. Our overall objectives were to (1) determine the adsorption behavior of four PFAS onto the binding surfaces of organoclay at different pHs; (2) measure isothermal adsorption of PFAS to the surfaces of the best hydrophobic clays and determine the best adsorption mode; (3) characterize the thermodynamics of the interaction of PFAS to the optimal organoclays; and (4) establish the proof-of-concept for the detoxification efficacy of the clays using *Hydra vulgaris* as a living model system.

## Materials and methods

### Organoclays and chemicals

The four organoclays are products of Bentonite Performance Mineral LLC, Halliburton (Houston, TX). The sodium bentonite is extracted from mines in Wyoming with an estimated cation exchange capacity equal to 75 cmol kg^−1^, external surface area of approximately 70 m^2^ g^−1^ and an average total surface area as high as 850 m^2^ g^−1^. The generic formula for these clays is (Na,Ca)_0.3_(Al,Mg)_2_Si_4_O_10_(OH)_2_·nH_2_O. The four organoclays are modified sodium bentonites manufactured by mixing a quaternary amine with the clay resulting in hydrophobic materials including ENVIRO-TROL™ (ENT), ORGANO-TROL™ (OT2), and AGRI-TROL™ (ATB and ATM)). Table S1 gives a summary of the physical properties of these organoclays. Ultrapure deionized water (18.2 MΩ) was used in this study and was produced with an Elga™ automated filtration system (Woodridge, IL). High-pressure liquid chromatography (HPLC)-grade acetonitrile and pH calibration buffers (4.0, 7.0, and 10.0) were sourced from VWR (Atlanta, GA, USA). PFAS (PFOA, GenX, PFBS, and PFOS) analytical standards were obtained from Sigma Aldrich (St. Louis, MO, USA).

### Hydrophobicity index of the organoclays

The hydrophobicity index by n-heptane-water vapor adsorption of the organoclays was evaluated using previous protocols (dos Reis et al. 2016; Rivenbark et al. 2022). Briefly, the organoclays were dried at 100 °C for 24 h, 300 mg of each organoclay dispensed into dry 10-mL beakers. Thereafter, they were placed in a sealed jar containing 60 mL of either n-heptane or water. After 24 h, the samples were taken out of the jar and weighed to calculate the mass absorption of n-heptane or water.

### Chemical analysis

Quantitative analysis of the four PFAS compounds was performed using a Waters Acquity Ultra Performance Liquid Chromatography system coupled with a tandem mass spectrometer (UPLC-MS/MS) equipped with a triple quadrupole detector following a previously established protocol (Wang et al. 2021; Oladele et al. 2025a, b; Xenophontos et al. 2025). Chromatographic separation was accomplished by an Acquity BEH C18 column (2.1 × 50 mm, 1.7 μm), which was maintained at a constant temperature (40 °C). The mobile phase was 20 mM ammonium acetate (eluent A) and acetonitrile (eluent B) carried at a flow rate of 0.30 mL/min using a gradient elution program: 10% eluent B (initial), 10–55% (0–1 min), 55–99% (1–9 min), 99% (9–10 min), and 99–10% (10–13 min) over a 13-min run time. Each injection contained 50 µL of each sample solution. Mass spectrometric detection was carried out with an electrospray ionization (ESI) source operated at negative mode and temperature source of 450 °C, with stray voltage of 4.5 kV. Analyte-specific precursor-to-product ion transitions (m/z) were monitored under multiple reaction monitoring (MRM) mode: 413 → 369 for PFOA, 499 → 80 for PFOA, 285 → 168.9 for GenX, and 298.9 → 80 for PFBS. Argon was utilized as both heater and nebulizer gas, while nitrogen served as the collision and curtain gas. System control for LC–MS/MS and data acquisition were done with Empower analyst software.

### Adsorption of PFAS at different pHs

In this study, adsorption behavior of the four PFAS (PFOA, GenX, PFBS, and PFOS) onto binding surfaces of the four organoclays and parent clay were examined at four different pH conditions (pH 2, 5, 7, 10) to mimic real-world pH ranges such as tap water (~ 7.5), bottled and distilled water (~ 5 to ~ 7), ocean, and freshwater (rivers, lakes, streams) (~ 6.5 to ~ 9.0) (USEPA 2025; WHO 2022; USGS n.d.). Briefly, stock solutions containing 10 ppm of each PFAS compound were prepared in ultrapure water which was adjusted to each pH condition. Subsequently, each organoclay was tested at 0.0005% (w/v) of 1 mL of PFAS solution. All samples were subjected to mechanical agitation at 1000 rpm for 2 h using an IKA® electric shaker (VIBRAX VXR basic, Werke, Germany) in a 37 °C incubator. After that, all samples were centrifuged for 5 min at 2000 × *g* to separate the adsorbent/PFAS complex from the solution. Supernatants of the controls and samples were analyzed using LC–MS/MS.

### Sorption isotherms at pH 2 and 7

The adsorption isotherms were carried out using a previously established protocol (Oladele et al. 2025a, b; Xenophontos et al. 2025). Briefly, each organoclay was added to a gradient solution of PFAS concentration ranging from 5 to 100%, at 0.0005% (w/v) of 1 mL of 10 ppm PFAS solution in pH 2 or 7. Controls included 1 mL of each sorbent solution, PFAS, and ultrapure water (either pH 2 or 7) as blank. All samples were subjected to mechanical agitation at 1000 rpm for 2 h using IKA® electric shaker (VIBRAX VXR basic, Werke, Germany) in a 37 °C incubator. After this, all samples were centrifuged for 5 min at 2000 × *g* and the supernatants were analyzed using a LC–MS/MS.

### Data analysis, calculations, and curve fitting

After the amount of unbound PFAS (PFOA, GenX, PFBS, and PFOS) in the solution was determined using LC-M/MS, the amount adsorbed by the sorbent materials was calculated as the difference between the test sample and the respective PFAS controls. The resulting data were expressed as mol/kg and plotted as isotherm plots using Table-curve 2D V. 5.01 (Systat Software, Inc., Palo Alto, CA, USA) and R programming language to evaluate the adsorption data and parameters (Yan et al. 2015; Gong and Rshiny 2020). This included the calculation of variables through non-linear regression analysis, and the selection of the best-fit adsorption model was based on the correlation coefficients (*r*^2^) and the distribution of the residuals from triplicate studies. The experimental data were interpreted using established adsorption isotherm models (Freundlich and Langmuir) (Grant and Phillips, 1998).

The Freundlich isotherm was used to describe the adsorption characteristics for a heterogeneous surface. The Freundlich model was represented by the following equation:

1 $$Freundlich\;model\;q\;=K_fC_w^{1/n}$$

K_f_ = Freundlich distribution constant, 1/*n* = degree of heterogenicity.

The Langmuir equation and functions:

2 $$Langmuir\;model\;q\:=Q_{max}\frac{K_dC_w}{1+K_dC_w}$$

where *C*_w_ = equilibrium concentration of PFAS (mol L^−1^), *K*_d_ = Langmuir distribution constant, *Q*_max_ = maximum binding capacity (mol kg^−1^), and *q* = the amount of PFAS adsorbed (mol kg^−1^).

The following equation was applied to determine *Ke°* from *K*_*d*_:

3 $$K_e^{{}^o}\frac{K_d{\lbrack adsorbate\rbrack}^o}\gamma$$

here, [adsorbate]° was the standard concentration of the adsorbate = 1 mol/L, K_e_° was the thermodynamic equilibrium constant, and *γ* was the coefficient of activity.

The free energy (*ΔG°*) and enthalpy (*ΔH*) for the adsorption process were calculated using the van’t Hoff and Gibbs free energy equations:

4 $$\triangle H=\frac{-\mathrm{RIn}(\frac{{\mathrm{K}}_{\mathrm{d}2}}{{\mathrm{K}}_{\mathrm{d}1}})}{\left(\frac1{{\mathrm{T}}_2}\right)-(\frac1{{\mathrm{T}}_1})}$$

5 $$\Delta G\:=\:\Delta G^\circ\:+\:RTInKe^\circ$$

where *R* (gas constant) = 8.314 J/mol/K, and *T* (absolute temperature) = 273 + *t* (°C). When *ΔG* is negative, the adsorption process is spontaneous and enhanced thermodynamically, thus proceeding in the forward direction (Ke° > 1). However, when it is positive, the adsorption process is not energetically favorable (Ke° < 1) and *ΔG* will be zero for an adsorption system at equilibrium.

### Ecotoxicological bioassay (*Hydra vulgaris*)

The *Hydra vulgaris* model reported by Oladele et al. (2025a, b) has been well established in our laboratory as a highly sensitive in vivo assay for environmental chemicals including PFAS. This living organism was used to investigate the detoxification efficacy of the organoclays in this study. Briefly, *Hydra vulgaris* obtained from Environmental Canada in Montreal were kept under controlled laboratory conditions at 18 °C. A detailed morphological scoring system on a scale of 1 to 10 was used to evaluate the various degrees of toxicity resulting from PFAS exposure. *Hydra vulgaris* were exposed to 25 ppm of PFAS and organoclays (individually) at three inclusion rates (0.1%, 0.2%, and 0.5%). Each experiment was carried out in three independent replicates from two independent hydra colonies. Morphological scores were recorded at intervals of 0, 4, 20, 28, 44, 68, and 92 h to monitor the progression of PFAS-mediated toxicity and to evaluate the protective effect offered by each of the organoclays.

### *Lemna minor*

Extensive earlier research has demonstrated that *Lemna minor* serves as an effective model organism for investigating toxicity of PFAS, pesticides, and other environmental contaminants (EPA 2012; Oladele et al. 2025a, b). In this study, *Lemna minor* were obtained from AquaHabit (England) and cultivated under controlled laboratory conditions. Plant growth was supported using a prepared Steinberg nutrient medium containing 0.66 mM KH_2_PO_4_, 3.46 mM KNO_3_, 4.03 μM EDTA, 1.94 μM H_3_BO_3_, 0.63 μM ZnSO_4_, 0.18 μM Na_2_MoO_4_, 0.41 mM MgSO_4_, 0.072 mM K_2_HPO_4_, 1.25 mM Ca(NO_3_)_2_, 2.81 μM FeCl_3_, and 0.91 μM MnCl_2_, adjusted to a pH of 5.5 ± 0.2 (Drost et al., 2007). Illumination was provided by cool white, fluorescent lamps delivering approximately 400 ft-c of light intensity, while the ambient temperature was maintained at 25 °C.

Two colonies of *Lemna minor*, each containing four fronds, were randomly selected and placed into individual wells of 24-well culture plates. The plates were incubated for 7 days with lids slightly loosened to allow gas exchange. For detoxification assessments, plants were subjected to 40 ppm PFAS and inclusion of SB and organoclays at 0.1 to 0.5% (w/v) for 7 days. Daily monitoring of frond number and surface area was carried out using NIH ImageJ software (Bethesda, MD). At the end of the exposure period, chlorophyll extraction was performed by homogenizing the plant material in 1.5 mL of 80% acetone. Following a 48-h incubation at − 4 °C, chlorophyll content was quantified using a UV–Vis scanning spectrophotometer (Shimadzu UV-1800, Kyoto, Japan).

### Statistical analysis

All experiments, including negative and blank controls, were performed in triplicate. Statistical analysis was conducted using a one-way ANOVA followed by Tukey’s post hoc test to evaluate differences between treatment groups. Results with *p* < 0.05 were considered statistically significant.

## Results

### Adsorption of PFOA at different pHs

**Fig. 1:**
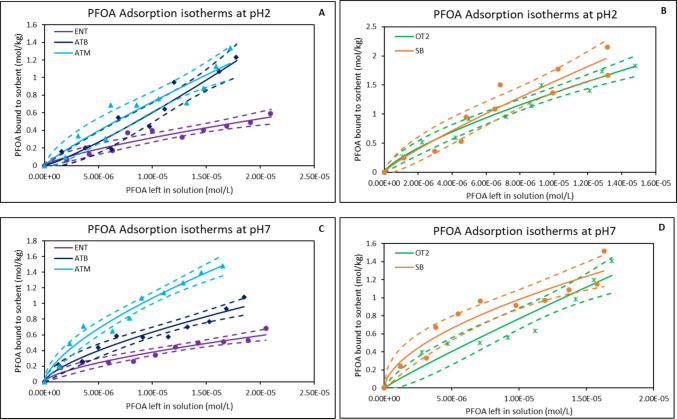
Freundlich isotherms showing adsorption of PFOA onto binding surfaces of organoclays at pH 2 (**A** & **B**) and pH 7 (**C** & **D**). ENT, ENVIRO-TROL; ATB, AGRI-TROL B; ATM, AGRI-TROL M; OT2, ORGANO-TROL; SB, sodium bentonite clay; The solid lines represent the adsorption isotherm plots based on the Langmuir model, while the dashed lines represent the 95% confidence band

The adsorption efficacy of PFOA onto surfaces of the organoclays: ENVIRO-TROL™ (ENT), ORGANO-TROL™ (OT2), and AGRI-TROL™ (ATB and ATM) was evaluated at various pH 2, pH 5, pH 7, and pH 10 to mimic environmental variabilities as shown in Table 1. Adsorption was highest at pH 2 with all the tested organoclays. OT2 demonstrated the maximum adsorption (78%) under acidic conditions, indicating favorable interactions and surface affinity at low pH. This high adsorption may be due to the high hydrophobicity of OT2 (Table S2). As pH increased to pH 5, there is a slight decrease in adsorption. At pH 7, adsorption varied markedly between the tested materials (organoclays and parent clay). OT2 demonstrated the highest binding capacity of 68%, followed by ATB with binding capacity of 61%; ET and ATM showed similar binding capacity (54%) while SB demonstrated the least binding (42%). At pH 10, adsorption performance significantly decreased in all tested materials. OT2 maintained the highest binding capacity of 49%, followed by ATM (47%), ENT, and ATB which showed the lowest binding capacity < 30%. All the organoclays showed good pH resilience between pH 2 and 7. Furthermore, all the organoclays demonstrated higher binding capacity than the parent clay at all pHs. In Fig. 1, adsorption isotherms were fitted to a Freundlich model with high correlation (*r*^2^ ≥ 0.90) indicating a good fit (Table 2). At pH 2 (Fig. 1 A and B), all tested materials exhibited a moderately high adsorption capacity for PFOA as shown by a Freundlich constant K_f_ with a magnitude of 10^3^ to 10^5^. ATB exhibited the highest capacity (4.89 × 10^5^ mol/kg). The ∆G values for all test materials ranged from − 25.87 to − 30.96 kJ/mol indicating that the adsorption of PFOA to the surface was spontaneous and in the forward direction. At pH 7 (Fig. 1 C and D), the adsorption behavior remained favorable across all sorbents with *r*^2^ values ranging from 0.91 to 0.97. OT2 demonstrated the highest K_f_ of 3.18 × 10^4^ followed by ATM (1.99 × 10^3^) and ATB (1.49 × 10^3^). The adsorption remained spontaneous with ∆G values ranging from − 26.85 to − 29.50 kJ/mol.

**Table 1:** Adsorption of four PFAS onto surfaces of organoclays at different pHs

Organoclays	pH 2	pH 5	pH 7	pH 10
**PFOA** (percentage adsorbed (%)				
ENT	50.37 ± 7.60	50.99 ± 8.81	54.88 ± 10.91	18.81 ± 1.14
ATB	57.46 ± 8.71*	52.25 ± 3.60	61.19 ± 13.13	29.11 ± 7.77
ATM	69.96 ± 7.10*	54.56 ± 4.02	54.31 ± 2.38*	47.03 ± 3.47*
OT2	78.47 ± 7.12*	54.10 ± 0.88	68.01 ± 12.59	49.01 ± 2.40*
SB	40.06 ± 4.69	41.64 ± 11.03	42.06 ± 5.37	24.80 ± 7.24
**PFOS** (percentage adsorbed (%)				
ENT	32.30 ± 8.38	34.17 ± 7.60*	16.21 ± 3.68	30.81 ± 1.78*
ATB	37.12 ± 7.36*	30.37 ± 1.50*	21.08 ± 5.85	22.60 ± 2.21*
ATM	43.73 ± 6.34*	32.74 ± 1.36*	26.74 ± 6.93	18.44 ± 4.78*
OT2	47.30 ± 7.72*	33.06 ± 1.46*	32.91 ± 6.20	20.43 ± 1.78*
SB	25.16 ± 1.17	18.79 ± 2.49	17.31 ± 2.94	3.96 ± 0.39
**GenX** (percentage adsorbed (%)				
ENT	30.28 ± 7.70	33.48 ± 7.98*	15.82 ± 7.63	11.95 ± 4.15*
ATB	37.60 ± 5.09*	27.74 ± 1.46	15.45 ± 5.55	32.48 ± 4.06*
ATM	43.23 ± 9.52*	32.14 ± 4.87*	13.67 ± 2.95	28.39 ± 2.20*
OT2	53.75 ± 9.42*	37.63 ± 8.27*	26.41 ± 10.47	30.76 ± 4.11*
SB	24.69 ± 6.45	21.53 ± 4.51	9.72 ± 2.09	8.45 ± 1.89
**PFBS** (percentage adsorbed (%)				
ENT	25.43 ± 1.72	23.25 ± 2.17*	18.55 ± 4.32	17.28 ± 0.86*
ATB	25.27 ± 6.80	17.05 ± 3.33	21.55 ± 6.53	32.42 ± 7.86*
ATM	40.34 ± 8.00*	20.19 ± 1.57*	20.94 ± 5.04	33.95 ± 0.04*
OT2	50.54 ± 10.86*	22.72 ± 2.78*	25.48 ± 7.04	33.15 ± 1.52*
SB	19.85 ± 5.96	15.60 ± 2.34	17.45 ± 3.21	11.63 ± 3.05

*ENT*, ENVIRO-TROL; *ATB*, AGRI-TROL B; *ATM*, AGRI-TROL M; *OT2*, ORGANO-TROL; *SB*, sodium bentonite clay

∗ Indicates a significant difference (*p *< 0.05) compared to SB

**Table 2:** Parameters and correlation coefficients from adsorption isotherms for the four PFAS

Organoclays	Adsorption parameters @ pH 2				Adsorption parameters @ pH 7			
	1/*n*	K_f_	∆G	*r*^**2**^	1/*n*	K_f_	∆G	*r*^2^
**PFOA**								
ENT	1.33	1.84 × 10^3^	− 26.43	0.92	1.56	6.16 × 10^2^	− 26.85	0.93
ATB	0.85	4.89 × 10^5^	− 28.74	0.92	1.48	1.49 × 10^3^	− 28.30	0.94
ATM	1.14	1.69 × 10^4^	− 29.03	0.90	1.53	1.99 × 10^3^	− 29.40	0.97
OT2	1.35	6.87 × 10^3^	− 30.23	0.96	1.08	3.18 × 10^4^	− 29.22	0.91
SB	1.17	2.84 × 10^4^	− 25.87	0.91	1.75	7.04 × 10^2^	− 29.50	0.91
**PFOS**								
ENT	0.77	6.81 × 10^5^	− 26.33	0.95	0.83	2.86 × 10^5^	− 26.34	0.93
ATB	1.25	2.33 × 10^3^	− 25.80	0.90	0.71	2.83 × 10^6^	− 26.51	0.96
ATM	0.69	4.14 × 10^6^	− 26.68	0.91	0.65	3.38 × 10^6^	− 23.73	0.93
OT2	0.78	2.07 × 10^6^	− 29.31	0.95	1.30	1.09 × 10^3^	− 24.48	0.90
SB	0.82	8.61 × 10^5^	− 29.03	0.96	1.30	1.30 × 10^3^	− 25.11	0.95
**GenX**								
ENT	1.01	5.87 × 10^4^	− 28.80	0.92	0.76	1.51 × 10^6^	− 28.00	0.91
ATB	0.93	8.35 × 10^4^	− 26.65	0.92	0.77	9.37 × 10^5^	− 27.26	0.93
ATM	1.51	1.39 × 10^3^	− 27.84	0.97	0.70	4.01 × 10^6^	− 27.86	0.91
OT2	1.29	6.80 × 10^4^	− 29.84	0.98	0.90	6.99 × 10^4^	− 25.97	0.92
SB	1.96	4.52 × 10^2^	− 29.31	0.93	2.76	2.50 × 10^0^	− 25.49	0.92
**PFBS**								
ENT	1.58	5.63 × 10^2^	− 26.46	0.97	0.77	1.81 × 10^5^	− 23.28	0.91
ATB	1.47	1.15 × 10^3^	− 26.77	0.97	0.67	1.78 × 10^6^	− 23.99	0.95
ATM	0.85	4.10 × 10^5^	− 28.39	0.90	0.65	1.12 × 10^6^	− 21.95	0.94
OT2	0.87	5.34 × 10^5^	− 29.64	0.92	0.68	2.26 × 10^6^	− 25.18	0.91
SB	0.88	3.96 × 10^5^	− 29.66	0.95	0.75	6.04 × 10^5^	− 25.43	0.98

*ENT*, ENVIRO-TROL; *ATB*, AGRI-TROL B; *ATM*, AGRI-TROL M; *OT2*, ORGANO-TROL; *SB*, sodium bentonite clay; *K*_*f*_, Freundlich distribution constant; *1/n*, degree of heterogenicity; *∆G*, Gibbs free energy (kJ/mol); *r*^*2*^, correlation coefficients

### Adsorption of PFOS at different pHs

**Fig. 2:**
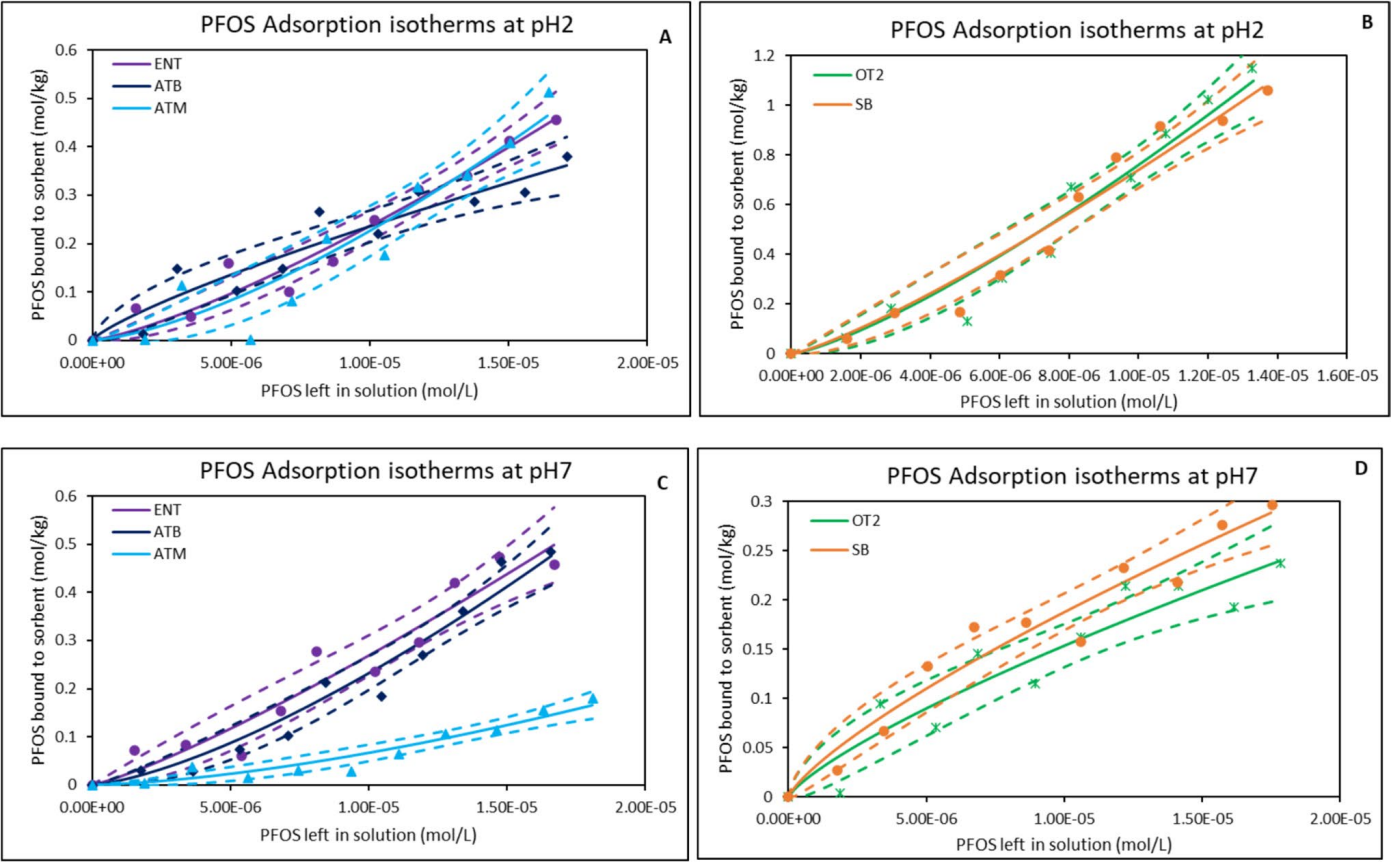
Freundlich isotherms showing adsorption of PFOS onto binding surfaces of organoclays at pH 2 (**A** & **B**) and pH 7 (**C** & **D**). ENT, ENVIRO-TROL; ATB, AGRI-TROL B; ATM, AGRI-TROL M; OT2, ORGANO-TROL; SB, sodium bentonite clay; The solid lines represent the adsorption isotherm plots based on the Langmuir model, while the dashed lines represent the 95% confidence band

At pH 2, OT2 exhibited the highest PFOS adsorption with approximately 47% adsorption. ATM followed by 44%, whereas ATB and ENT showed moderate adsorption (37% and 32% respectively). SB demonstrated the least adsorption (25%) (Table 1). At pH 5, PFOS adsorption was reduced in all tested materials except with ENT. All the organoclays demonstrated similar PFOS binding capacities ranging from 30 to 34%. Again, SB showed the least adsorption (19%). At neutral pH, all the organoclays showed adsorption ranging from 16 to 32%. At alkaline conditions (pH 10), PFOS adsorption ranged from 18 to 31%. The organoclays (especially OT2 and ATM) demonstrated higher PFOS adsorption than SB across all pHs, indicating that the amendment enhanced their binding capacity. The adsorption isotherms for PFOS binding onto the surfaces of the organoclays at pH 2 and pH 7, respectively, are depicted in Fig. 2. The PFOS isotherms for all the tested materials followed a Freundlich model with high correlation (*r*^2^ ≥ 0.90) indicating a good fit (Table 2). At pH 2 (Fig. 2 A and B), the tested materials showed favorable adsorption behavior (1/*n* < 1) suggesting that as the concentration increases, the adsorption also increases. This also indicated a heterogeneous surface and strong PFOS-sorbent interactions. The organoclays exhibited high K_f_ in the magnitude of 10^3^ to 10^6^. ATM and OT2 exhibited the highest capacity of 4.14 × 10^6^ and 2.07 × 10^6^ mol/kg respectively. The ∆G values (− 25.80 to − 29.31 kJ/mol) indicate that the adsorption of PFOS was spontaneous at pH 2. Furthermore, at pH 7 (Fig. 2 C and D), the adsorption behavior remained favorable across all sorbents with 1/*n* values ranging between 0.67 and 1.30. Also, *r*^2^ values were between 0.90 and 0.96. ATM demonstrated the highest K_f_ of 3.38 × 10^6^, followed closely by ATB (2.83 × 10^6^). The spontaneity of PFOS adsorption was maintained and the ∆G values ranged from − 25.11 to − 27.24 kJ/mol.

### Adsorption of GenX at different pHs

**Fig. 3:**
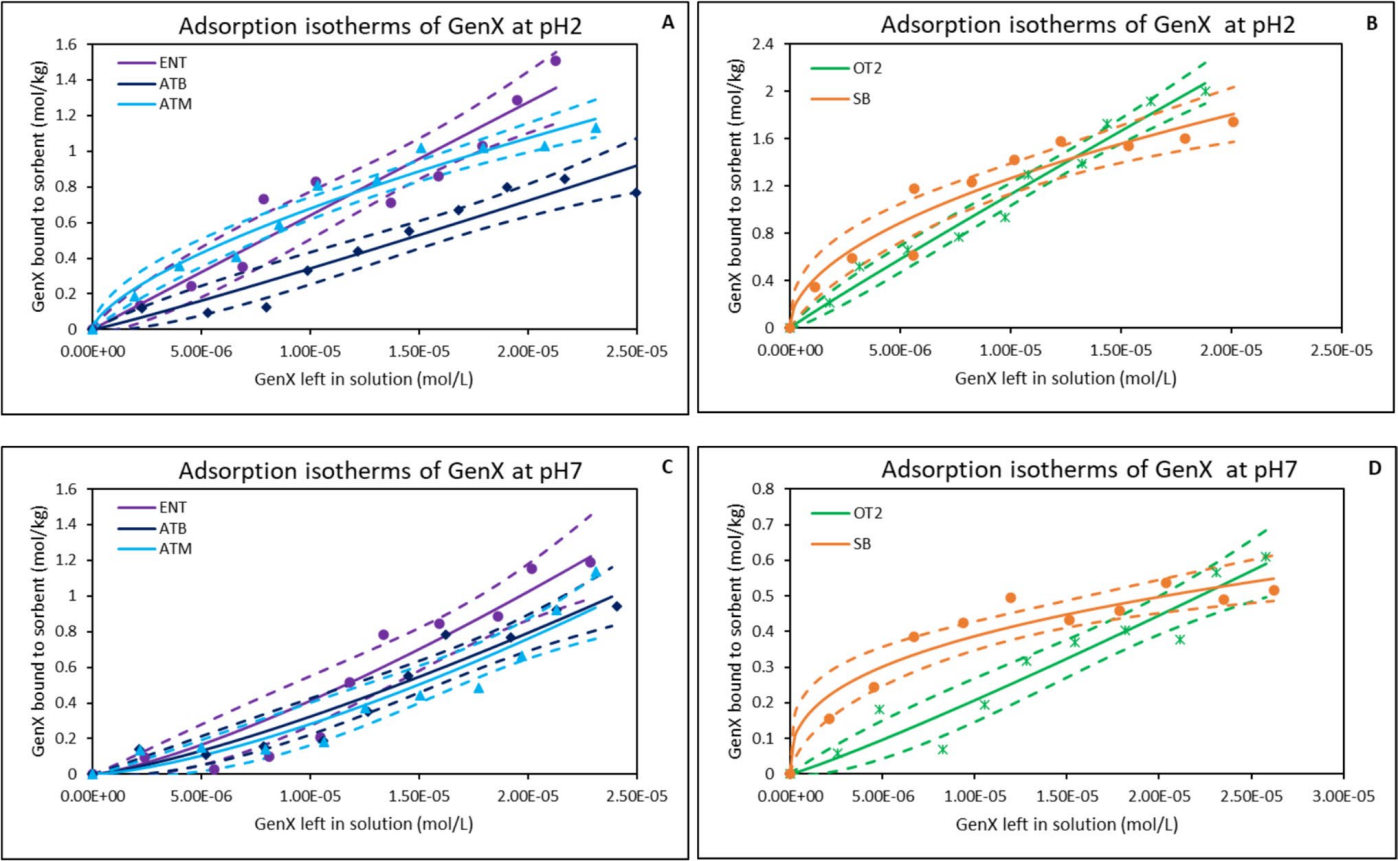
Freundlich isotherms showing adsorption of GenX onto binding surfaces of organoclays at pH 2 (**A** & **B**) and pH 7 (**C** & **D**). ENT, ENVIRO-TROL; ATB, AGRI-TROL B; ATM, AGRI-TROL M; OT2, ORGANO-TROL; SB, sodium bentonite clay; The solid lines represent the adsorption isotherm plots based on the Langmuir model, while the dashed lines represent the 95% confidence band

The result of the adsorption capacity of each of the organoclays for GenX was assessed across four pH levels and is presented in Table 1. At pH 2, among the tested organoclays, OT2 exhibited the highest adsorption capacity (54%), followed by ATM (43%), ATB (38%), and ENT (30%), compared with parent clay SB (25%). As the pH increased to 5, adsorption efficiencies declined across all the sorbent materials. OT2 maintained the highest level (38%) and the remaining organoclays ranged between 28 and 33%. At neutral pH (7) and alkaline pH (10), all organoclays exhibited reduced adsorption. Adsorption percentages across all materials fell between 12 and 33%. Under acidic conditions (Fig. 3 A and B), ATB and OT2 emerged as the most effective organoclay with highest K_f_ of 8.35 × 10^4^ and 6.89 × 10^4^ respectively, followed by ENT (5.87 × 10^4^). The ∆G values (− 26.65 to − 29.84 kJ/mol) showed that the adsorption of GenX is thermodynamically favorable. SB showed the least K_f_ of 4.52 × 10^2^ suggesting high affinity but limited capacity. At neutral pH condition (Fig. 3 C and D), the adsorption performance shifted. All the organoclays demonstrated favorable adsorption behavior (1/*n* < 1). ATM exhibited the highest K_f_ (4.01 × 10^6^), followed by ENT (1.51 × 10^6^). Negative ∆G values for all the sorbents indicated that adsorption was spontaneous.

### Adsorption of PFBS at different pHs

**Fig. 4:**
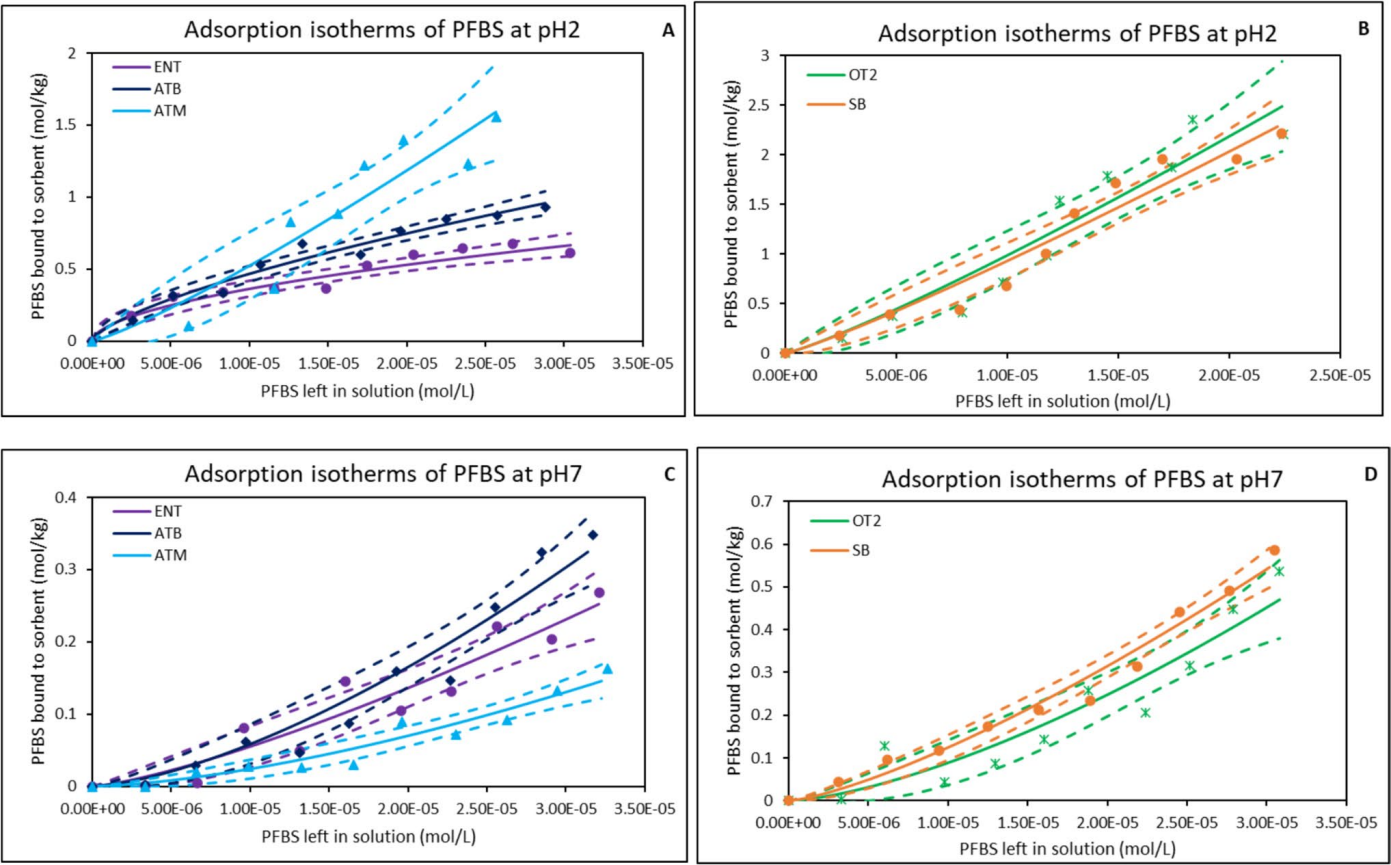
Freundlich isotherms showing adsorption of PFBS onto binding surfaces of organoclays at pH 2 (**A** & **B**) and pH 7 (**C** & **D**). ENT, ENVIRO-TROL; ATB, AGRI-TROL B; ATM, AGRI-TROL M; OT2, ORGANO-TROL; SB, sodium bentonite clay; The solid lines represent the adsorption isotherm plots based on the Langmuir model, while the dashed lines represent the 95% confidence band

Table 1 shows results of the adsorption of PFBS onto the surfaces of the tested sorbent materials. At acidic conditions (pH 2), OT2 exhibited the highest adsorption capacity (51%), followed by ATM (40%) while ENT and ATB exhibited similar adsorption capacity of 25%. SB showed the least adsorption (20%). As the pH increased to 5, adsorption declined across all the organoclays ranging from 17 to 23%. The range of adsorption capacities observed at pH 7 was similar to that of pH 5; however, OT2 had the highest adsorption (25%). Interestingly, all the organoclays demonstrated higher adsorption capacity for PFBS at pH 10 versus pH 5 and pH 7, except ENT. They showed adsorption capacity of 33% each. At pH 2 (Fig. 4 A and B), among the organoclays, OT2 demonstrated the highest adsorption capacity with K_f_ of 5.34 × 10^5^ followed by ATM (4.10 × 10^5^), and ATB (1.15 × 10^3^). OT2, ATM, and SB showed favorable adsorption behavior with 1/*n* ranging between 0.85 and 0.88. The ∆G values (− 26.46 to − 29.66 kJ/mol) suggested strong spontaneity. At pH 7 (Fig. 4 C and D), all sorbent materials exhibited favorable adsorption with 1/*n* values less than 1. OT2 emerged as the best sorbent with K_f_ of 2.26 × 10^6^ followed by ATB (1.78 × 10^6^), and ATM (1.12 × 10^6^). The ∆G values (− 21.95 to − 25.43 kJ/mol) suggested that they retained strong thermodynamic spontaneity.

### Toxic effects of PFAS on *Hydra vulgaris* and the protective role of organoclays

**Fig. 5:**
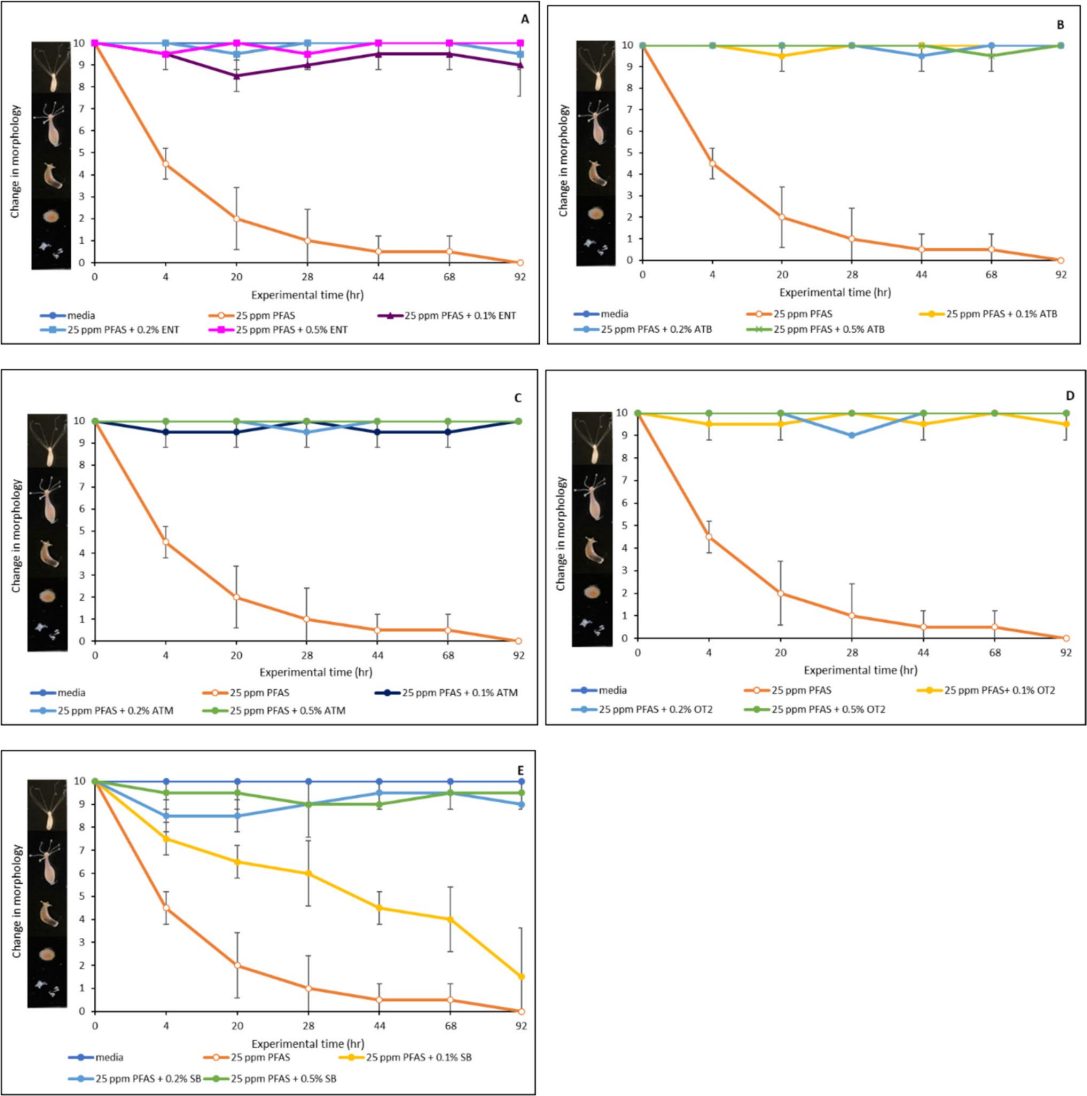
Protective effect of organoclays on PFAS-induced toxicity in *Hydra vulgaris.* **A** ENT, **B** ATB, **C** ATM, **D** OT2, and **E** SB clays. ENT, ENVIRO-TROL; ATB, AGRI-TROL B; ATM, AGRI-TROL M; OT2, ORGANO-TROL; SB, sodium bentonite clay

The data for the in vivo assessment of PFAS toxicity in *Hydra vulgaris* and the protection offered by the organoclays are presented in Fig. 5. PFAS (25 ppm mixture of PFOA, PFOS, PFBS, and GenX) caused 90% morphological damage to the hydra after 28 h of exposure and its toxicity increased to 100% damage after 44 h of exposure. The hydra were completely disintegrated and dead after 68 h of exposure. However, inclusion of the organoclays demonstrated significant protection at the various inclusion rates (0.1 to 0.5%) used in this study (Fig. 5A–D). At only 0.1% inclusion, the organoclays resulted in 95 to 100% protection. Remarkably, all of the organoclays resulted in complete protection at the 0.2% inclusion rate. In contrast, the parent clay only demonstrated limited protection at the same inclusion rates (Fig. 5E), and this observation showed the advantage of the organoclays over the parent bentonite.

### Toxic effects of PFAS on *Lemna minor* and the protective role of organoclays

**Fig. 6:**
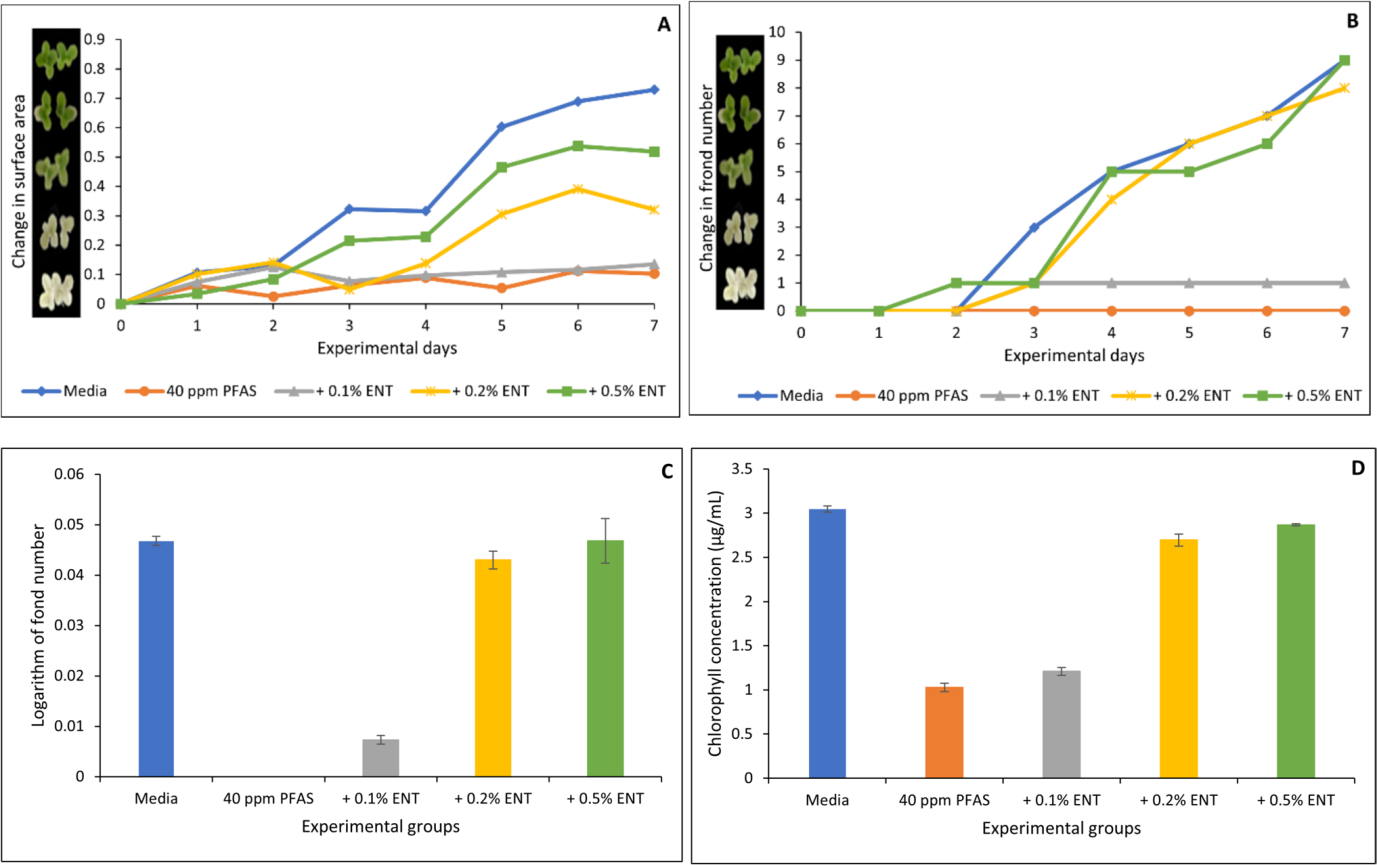
Protection of *Lemna minor* from PFAS toxicity by ENVIRO-TROL (ENT) based on surface area (**A**), frond number (**B**), growth rate at day 7 (**C**), and chlorophyll content (**D**). * indicates a significant difference (*p* < 0.05) compared to the vehicle control group

**Fig. 7:**
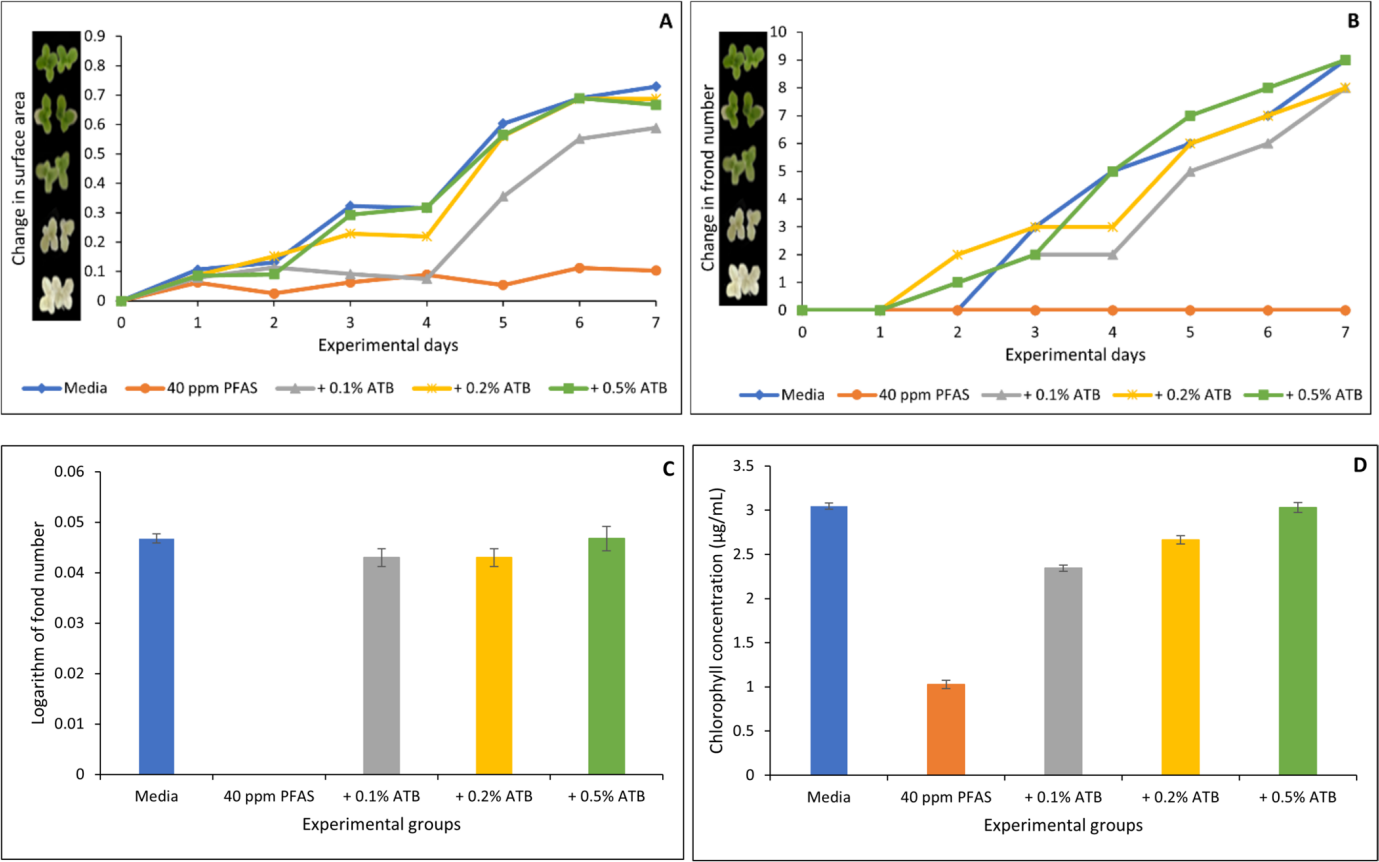
Protection of *Lemna minor* from PFAS toxicity by AGRI-TROL B (ATB) based on surface area (**A**), frond number (**B**), growth rate at day 7 (**C**), and chlorophyll content (**D**). * indicates a significant difference (*p* < 0.05) compared to the vehicle control group

**Fig. 8:**
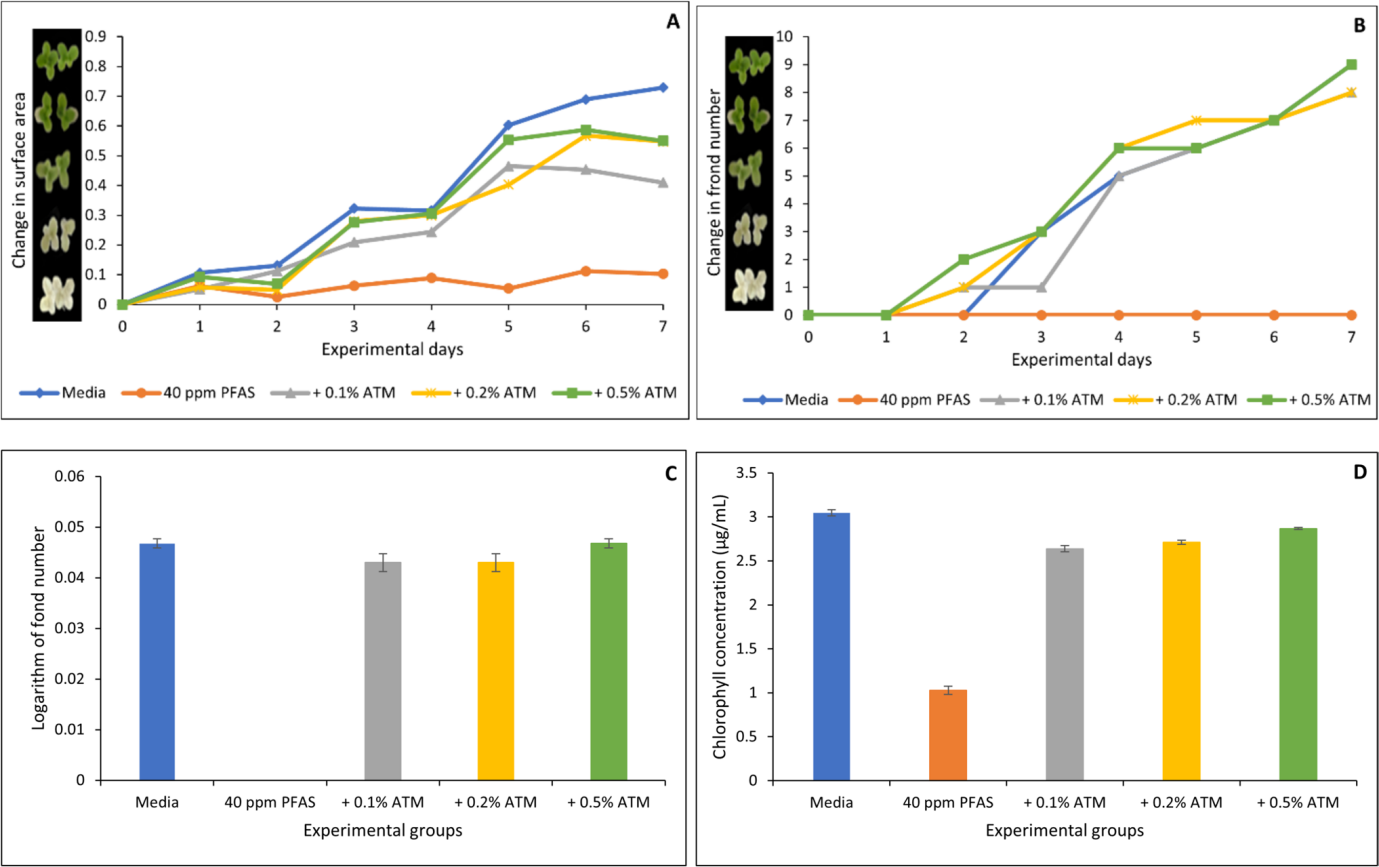
Protection of *Lemna minor* from PFAS toxicity by AGRI-TROL M (ATM) based on surface area (**A**), frond number (**B**), growth rate at day 7 (**C**), and chlorophyll content (**D**). * indicates a significant difference (*p* < 0.05) compared to the vehicle control group

**Fig. 9:**
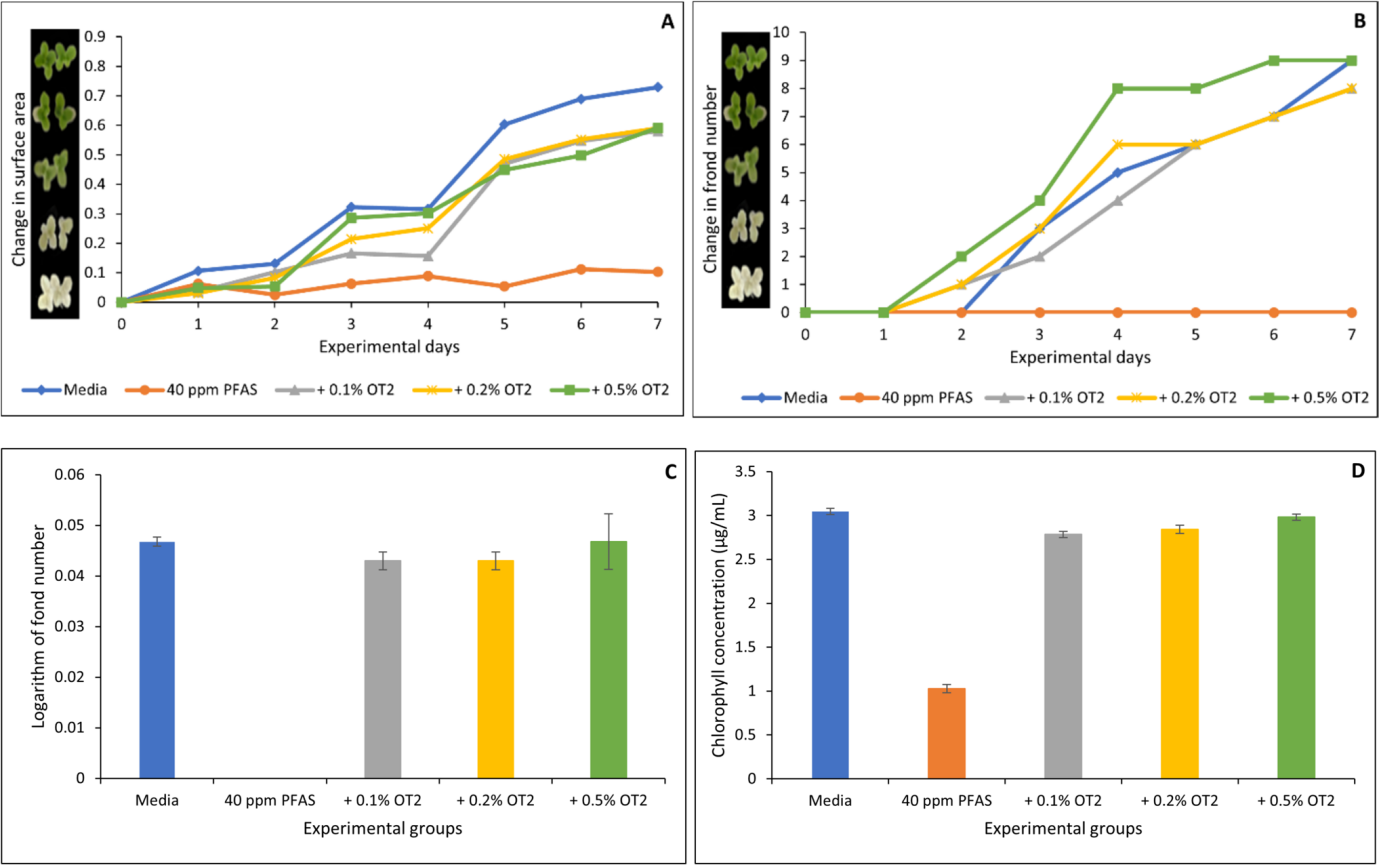
Protection of *Lemna minor* from PFAS toxicity by ORGANO-TROL (OT2) based on surface area (**A**), frond number (**B**), growth rate at day 7 (**C**), and chlorophyll content (**D**). * indicates a significant difference (*p* < 0.05) compared to the vehicle control group

**Fig. 10:**
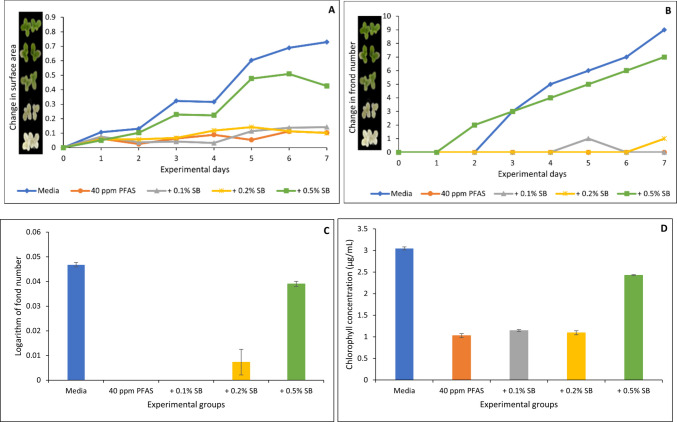
Protection of *Lemna minor* from PFAS toxicity by sodium bentonite (SB) based on surface area (**A**), frond number (**B**), growth rate at day 7 (**C**), and chlorophyll content (**D**). * indicates a significant difference (*p* < 0.05) compared to the vehicle control group

Figures 6, 7, 8, 9 and 10 show both the toxicological impact of PFAS exposure on *Lemna minor* and the protection offered by the organoclays. At 40 µg/mL, PFAS significantly inhibited *Lemna minor* growth compared to the control group, resulting in reductions of approximately 86%, 100%, 100%, and 66% in surface area (Fig. 6 A), frond number (Fig. 6 B), growth rate (logarithmic rate; Fig. 6 C), and chlorophyll content (Fig. 6 D), respectively. However, treatment with the four organoclays markedly prevented PFAS-induced toxicities. At 0.2% and 0.5% inclusion, ENT offered 60 to 100% protection (Fig. 6 A through D). ATB, ATM, and OT2 demonstrated 80 to 100% protection (Figs. 7, 8, 9). In contrast, the parent clay only showed protection at the highest inclusion rate (0.5%) (Fig. 10). This observation confirms the efficacy of the organoclays over the parent bentonite.

## Discussion

This study examined the adsorption efficacy of four PFAS onto the binding surfaces of four commercially available organoclays with the aim of characterizing the most effective PFAS binders. The data from this study demonstrated a clear pH-dependent pattern in adsorption efficiency, where acidic conditions (pH 2 and pH 5) supported higher PFAS (PFOA, PFOS, PFBS, and GenX) adsorption across all tested materials. This observation aligns with our earlier findings which suggested an enhanced electrostatic attraction between protonated clay surfaces (at acidic pH) and those surfaces that had been previously modified with positively charged quaternary amines that react electrostatically with the negatively charged head-groups of PFAS (Wang et al. 2021). OT2 and ATM consistently exhibited strong adsorption capacity among the tested organoclays under acidic, neutral, and alkaline conditions. This indicates that OT2 and ATM possess important hydrophobic functional groups, high surface area, and favorable charge distribution that enhanced PFAS binding. OT2 maintained high adsorption performance across all pH levels, suggesting the clay’s potential robustness in variable environmental conditions. Importantly, unmodified SB consistently demonstrated the least amount of adsorption of all the four PFAS (PFOA, GenX, PFBS, and PFOS), which underscores limited capacity for PFAS removal and the importance of enhanced hydrophobicity and positively charged clay surfaces for optimal efficacy.

These findings are relevant for environmental applications of organoclays in PFAS-contaminated water and soil. OT2 and ATM, which maintain high adsorption capacity for all four PFAS across a wide range of pH, may offer sustainable applications as PFAS adsorbents in rural, urban, and agricultural zones.

Additionally, the use of isothermal adsorption models in this investigation provided a mechanistic framework for predicting PFAS binding behavior which can help with the future design of tailored sorbents with enhanced capacity and selectivity. The adsorption isotherms for PFOA and PFOS showed strong affinity and capacity principally at acidic pH. All the organoclays, particularly OT2 and ATM demonstrated high adsorption capacities and favorable thermodynamics at both pH 2 and pH 7. This is consistent with PFOS’s strong surface activity and hydrophobicity (Fatima et al. 2025). Anionic PFAS, including PFOS and PFOA which have –SO_3_H and –COOH functional groups, respectively have been reported to bind surfaces of sorbents via anion and ligand exchange mechanisms (Wang et al. 2012). Furthermore, PFAS adsorption in soil is boosted through the formation of complexes between the hydroxyl bonds of aluminum and iron (hydro) oxides present in soils (Li et al. 2019).

The strong adsorption behavior of both PFOA and PFOS could be due to hydrophobic interactions with the lipophilic components in the organoclays. This aligns with the previous reports that long-chain PFAS (such as PFOS and PFOA) are principally adsorbed through hydrophobic interactions (Gagliano et al. 2020). Likewise, sorbents with amine groups have shown to typically have higher adsorption capabilities for PFOS and PFOA (Du et al. 2014). The shift in the adsorption performance of the organoclays across the pH conditions in this study further demonstrated the impact of electrostatic interactions in the adsorption process. According to Du et al. (2014), anionic PFAS interacts electrostatically with positively charged sorbent materials.

The short-chain PFAS (PFBS and GenX) showed variable and lower adsorption compared to the long-chained PFAS. GenX, a replacement for PFOA, has presented unique challenges for adsorption due to its lower hydrophobicity and smaller molecular size. Studies have shown that GenX is not as effectively adsorbed by activated carbon compared to PFOA with very low removal efficacy (Zhu et al. 2022). Importantly, all the tested materials in this study showed enhanced GenX adsorption at acidic conditions. Similarly, PFBS has also been reported to have low adsorption due to shorter chain length and reduced hydrophobicity. However, in this study, PFBS was effectively adsorbed by OT2 and ATM.

Interestingly, the adsorption isotherms for the four PFAS onto the organoclays and parent clay demonstrated Gibbs free energy (∆G) values ≥ − 20 kJ/mol. This suggested that the adsorption reaction was energetically favorable. The values fall within a range classically connected to strong physisorption or weak chemisorption, which indicate the possibility of specific interactions such as electrostatic interactions and ion exchange. This is further supported by the presence of a positively charged functional group (-NR^+^_4_) on the modified surfaces of the organoclays. These are stable to pH change and capable of participating in significant interactions with PFAS.

*Hydra vulgaris* have been well established as toxin-sensitive living organisms and used as an in vivo model to (a) confirm the toxicity of a wide range of environmental chemicals including PFAS; (b) validate in vitro results; and (c) confirm the safety of new adsorbent materials and their ability to protect against the toxicity of diverse chemicals. In this study, hydra exposed to 25 ppm of PFAS exhibited a range of toxic responses which led to severe and irreversible morphological damage and ultimately death. This finding was consistent with previous reports by Oladele et al. (2025a, b) which documented complete disintegration of *Hydra vulgaris* following exposure to PFAS mixtures at concentrations greater than 20 ppm. Importantly, the inclusion of the parent clay and organoclays did not cause significant toxicity to hydra. The organoclays provided remarkable protection from PFAS toxicity. In particular, all of the organoclays demonstrated complete protection at low inclusion rates. This protection against PFAS toxicity was offered over the 92 h exposure period used in this study. The model provides valuable insight into PFAS binding and supports the potential of these organoclays to serve as toxin binders in water and soil.

Furthermore, *Lemna minor* is an aquatic plant which serves as a model organism for assessing waterborne contaminants due to its rapid growth and sensitivity to toxic pollutants (EPA 2012). PFAS exposure to *Lemna minor* in this study showed a significant reduction in growth rate, chlorophyll content, and fond number. This agreed with the previous findings of Noori et al. (2025) who reported that PFOA exposure to *Lemna minor* for 14 days resulted in significant chlorosis and a reduction in chlorophyll pigment indicating impaired photosynthetic capacity. Furthermore, they reported significant increase in nuclear size, while essential nutrient concentrations such as potassium, copper, and iron were decreased, potentially disrupting metabolic processes (Noori et al. 2025). Similarly, PFAS exposure induced oxidative stress in *Lemna minor* (Gonzales, et al. 2023; Zhang and Liang 2021). The protective effect from toxicity observed in *L. minor* can be attributed to the high affinity of the organoclays for PFAS. The organoclays possess modified interlayer spaces and surface functionalities that enhance adsorption of the four PFAS, thereby reducing their concentration in the aqueous phase (Oladele et al. 2025a, b). This mechanism likely explains the improved physiological parameters in *Lemna minor* exposed to PFAS in the presence of organoclays, such as restoring chlorophyll levels and preserving frond integrity. These findings align with prior studies on clay-based sorbents for organic pollutants, extending their applicability to persistent fluorinated contaminants (Wang et al. 2021; Oladele et al. 2024a, b).

## Conclusion

Data from this study showed that all the organoclays effectively adsorbed the two classes of PFAS (short and long chain) at both normal and extreme environmental pH conditions. Mechanisms for this adsorption are most likely associated with strong interactions of PFAS at organophilic binding sites containing hydrophobic side chains, electrostatic interactions with the quaternary amine functional group, and formation of stable complexes. This study also established that the adsorption processes followed the Freundlich model and were thermodynamically favored. The organoclays significantly neutralized PFAS-induced toxicity in sensitive *Hydra vulgaris* and *Lemna minor* and reduced the PFAS level in their aqueous media. This implied that these organoclays can be packed into permeable reactive barriers (PRBs) to trap PFAS during groundwater treatment or as in situ capping materials for sediment remediation in lakes and rivers to sequester PFAS. Moreover, these organoclays can be incorporated into filtration systems for removal of PFAS during wastewater treatment. Embedding these organoclays into landfill liners, caps to prevent off-gassing, or polymer membranes could also facilitate selective sorption of PFAS and prevent toxic leachates into the urban, rural, and agricultural environment.

## Supplementary Information

Below is the link to the electronic supplementary material.

ESM1 (DOCX 18.2 KB)

## Data Availability

The authors declare that the data supporting the findings of this study are available within the paper and its supplementary files.

## References

[CR1] Arvaniti OS, Stasinakis AS (2015) Review on the occurrence, fate and removal of perfluorinated compounds during wastewater treatment. Sci Total Environ 524:81–92 25889547 10.1016/j.scitotenv.2015.04.023

[CR2] Bolan N, Sarkar B, Yan Y, Li Q, Wijesekara H, Kannan K, Tswang DCW, Schauerte M, Bosch J, Noll H, Ok YS, Scheckel K, Kumpiene J, Gobindlal K, Kah M, Sperry J, Wang H, Tsang YF, Hou D, Rinklebe J (2021) Remediation of poly- and perfluoralkyl substances (PFAS) contaminated soils – to mobilize or to immobilize or to degrade? J Hazard Mater 401:123892. 10.1016/j.jhazmat.2020.123892 33113753 10.1016/j.jhazmat.2020.123892 PMC8025151

[CR3] Buck RC, Franklin J, Berger U, Conder JM, Cousins IT, De Voogt P, Jensen AA, Kannan K, Mabury SA, Leeuwen SPJ (2011) Perfluoroalkyl and polyfluoroalkyl substances in the environment: terminology, classification, and origins. Integr Environ Assess Manag 7:513–541 21793199 10.1002/ieam.258 PMC3214619

[CR4] Chen Z, Li C, Gao J, Dong H, Chen Y, Wu B, Gu C (2020) Efficient reductive destruction of perfluoroalkyl substances under self-assembled micelle confinement. Environ Sci Technol 54:5178–5185 32062968 10.1021/acs.est.9b06599

[CR5] Christensen KY, Raymond M, Blackowicz M, Liu Y, Thompson BA, Anderson HA, Turyk M (2017) Perfluoroalkyl substances and fish consumption. Environ Res 154:145–151 28073048 10.1016/j.envres.2016.12.032

[CR6] Costello MCS, Lee LS (2020) Sources, fate, and plant uptake in agricultural systems of per- and polyfluoroalkyl substances. Curr Pollut Rep 1–21

[CR7] Crawford NM, Fenton SE, Strynar M, Hines EP, Pritchard DA, Steiner AZ (2017) Effects of perfluorinated chemicals on thyroid function, markers of ovarian reserve, and natural fertility. Reprod Toxicol 69:53–59 28111093 10.1016/j.reprotox.2017.01.006 PMC5690561

[CR8] Domingo JL, Nadal M (2019) Human exposure to per-and polyfluoroalkyl substances (PFAS) through drinking water: a review of the recent scientific literature. Environ Res 177:108648 31421451 10.1016/j.envres.2019.108648

[CR9] dos Reis GS, Bin Mahbub MK, Wilhelm M, Lima EC, Sampaio CH, Saucier C, Dias SLP (2016) Activated carbon from sewage sludge for removal of sodium diclofenac and nimesulide from aqueous solutions. Korean J Chem Eng 33:3149–3161. 10.1007/s11814-016-0194-3

[CR10] Drost W, Matzke M, Backhaus T (2007) Heavy metal toxicity to lemna minor: studies on the time dependence of growth inhibition and the recovery after exposure. Chemosphere 67:36–43 17157350 10.1016/j.chemosphere.2006.10.018

[CR11] Du Z, Deng S, Bei Y, Huang Q, Wang B, Huang J, Yu G (2014) Adsorption behavior and mechanism of perfluorinated compounds on various adsorbents—a review. J Hazard Mater 274:443–454 24813664 10.1016/j.jhazmat.2014.04.038

[CR12] EPA – United States Environmental Protection Agency (2021) Basic Information on PFAS. www.epa.gov. Accessed 7 Apr 2021

[CR13] EPA United States Environmental Protection Agency EPA (2012) Ecological effects test guidelines OCSPP 850.4400: aquatic plant toxicity test using Lemna spp. EPA 712-C-008 January 2012. 1–24

[CR14] Fatima M, Kelso C, Hai F (2025) Perfluorooctanoic acid (PFOA) and perfluorooctanesulfonic acid (PFOS) adsorption onto different adsorbents: a critical review of the impact of their chemical structure and retention mechanisms in soil and groundwater. Water 17(9):1401. 10.3390/w17091401

[CR15] Gagliano E, Sgroi M, Falciglia PP, Vagliasindi FG, Roccaro P (2020) Removal of poly-and perfluoroalkyl substances (PFAS) from water by adsorption: role of PFAS chain length, effect of organic matter and challenges in adsorbent regeneration. Water Res 171:115381 31923761 10.1016/j.watres.2019.115381

[CR16] Garg S, Kumar P, Mishra V, Guijt R, Singh P, Dum’ee LF, Sharma RS (2020) A review on the sources, occurrence and health risks of per− /poly-fluoroalkyl substances (PFAS) arising from the manufacture and disposal of electric and electronic products. J Water Process Eng 38:101683

[CR17] Ghisi R, Vamerali T, Manzetti S (2019) Accumulation of perfluorinated alkyl substances (PFAS) in agricultural plants: a review. Environ Res 169:326–341 30502744 10.1016/j.envres.2018.10.023

[CR18] Gong J, Rshiny (2020) Available online: https://jgong9.shinyapps.io/chemistry_app/. Accessed 30 Sept 2023

[CR19] Gonzales AK, Donaher SE, Wattier BD, Martinez NE (2023) Exposure of *lemna minor* (common duckweed) to mixtures of uranium and perfluorooctanoic acid (PFOA). Environ Toxicol Chem 42:2412–2421. 10.1002/etc.5720 37477461 10.1002/etc.5720

[CR20] Kabore HA, Duy SV, Munoz G, M´eit´e L, Desrosiers M, Liu J et al (2018) Worldwide drinking water occurrence and levels of newly-identified perfluoroalkyl and polyfluoroalkyl substances. Sci Total Environ 616:1089–100 29100694 10.1016/j.scitotenv.2017.10.210

[CR21] Lee Y, Lo S, Kuo J, Hsieh C (2012) Decomposition of perfluorooctanoic acid by microwaveactivated persulfate: effects of temperature, pH, and chloride ions. Front Environ Sci Eng 6:17–25

[CR22] Li Y, Fletcher T, Mucs D, Scott K, Lindh CH, Tallving P, Fletcher T, Jakobson K (2018) Half-lives of PFOS, PFHxS and PFOA after end of exposure to contaminated drinking water. Occup Environ Med 75:46–51 29133598 10.1136/oemed-2017-104651 PMC5749314

[CR23] Li F, Fang X, Zhou Z, Liao X, Zou J, Yuan B, Sun W (2019) Adsorption of perfluorinated acids onto soils: kinetics, isotherms, and influences of soil properties. Sci Total Environ 649:504–514 30176462 10.1016/j.scitotenv.2018.08.209

[CR24] Liew Z, Goudarzi H, Oulhote Y (2018) Developmental exposures to perfluoroalkyl substances (PFASs): an update of associated health outcomes. Curr Environ Health Rep 5:1–19 29556975 10.1007/s40572-018-0173-4 PMC6348874

[CR25] Liu Y, D’Agostino AL, Qu G, Jiang G, Martin JWM (2019) High-resolution mass spectrometry (HRMS) methods for nontarget discovery and characterization of poly and per-fluoralkyl substances (PFASs) in environmental and human samples. TrAC Trends Anal Chem 121:115420

[CR26] Mukhopadhyay R, Sarkar B, Palansooriya KN, Dar JY, Bolan NS, Parikh SJ, Sonne C, Ok YS (2021) Natural and engineered clays and clay minerals for the removal of poly- and perfluoroalkyl substances from water: state-of-the-art and future perspectives. Adv Colloid Interface Sci 297:102537. 10.1016/j.cis.2021.102537 34624725 10.1016/j.cis.2021.102537

[CR27] Navarro I, de la Torre A, Sanz P, Pro J, Carbonell G, de los Angeles’ Martínez M (2016) Bioaccumulation of emerging organic compounds (perfluoroalkyl substances and halogenated flame retardants) by earthworm in biosolid amended soils. Environ Res 149:9–32 10.1016/j.envres.2016.05.004 27174781

[CR28] Noori A, Corbelli L, Lincoln E, Thomas S, Jones J, Nason SL, White JC, Lewis R, Haynes CL (2025) Phytotoxicity and phytoremediation potential of *Lemna minor* exposed to perfluorooctanoic acid. Frontiers in Plant Science 15:1493896. 10.3389/fpls.2024.1493896 39931343 10.3389/fpls.2024.1493896 PMC11807973

[CR29] OECD/UNEP Global PFC Group (2013) United Nations Environment Programme: synthesis paper on per- and polyfluorinated chemicals (PFCs). Environment, Health and Safety, Environment Directorate, OECD. IOMC Inter-Organization Program. Sound Manag Chem 1–58

[CR30] Oladele JO, Wang M, Rivenbark KJ, Phillips TD (2024) Application and efficacy of beidellite clay for the adsorption and detoxification of deoxynivalenol (vomitoxin). Emerging contaminants 10(4):100390. 10.1016/j.emcon.2024.100390 40276486 10.1016/j.emcon.2024.100390 PMC12021442

[CR31] Oladele JO, Wang M, Xenophontos X, Kendall L, Tamamis P, Phillips TD (2024a) Chlorophyll-amended organoclays for the detoxification of ochratoxin A. Toxins 16(11):1–32. 10.3390/toxins16110479 10.3390/toxins16110479 PMC11598794 39591234

[CR32] Oladele JO, Xenophontos X, Elizondo GM III, Daasari Y, Wang M, Tamamis P, Johnson NM, Phillips TD (2025a) Green-engineered montmorillonite clays for the adsorption, detoxification, and mitigation of aflatoxin B1 toxicity. Toxins 17(3):131. 10.3390/toxins17030131 10.3390/toxins17030131 PMC11945334 40137904

[CR33] Oladele JO, Xenophontos X, Wang M, Tamamis P, Phillips TD (2025b) Adsorption of per- and polyfluoroalkyl substances by edible nutraceutical-amended montmorillonite clays: in vitro, in vivo and in silico enterosorption strategies. Water Air Soil Pollut 236(293):1–25 10.1007/s11270-025-07930-2 PMC11971225 40190788

[CR34] Rivenbark KJ, Wang M, Lilly K, Tamamis P, Phillips TD (2022) Development and characterization of chlorophyll-amended montmorillonite clays for the adsorption and detoxification of benzene. Water Res 221:118788. 10.1016/j.watres.2022.118788 35777320 10.1016/j.watres.2022.118788 PMC9662585

[CR35] Sarkar B, Rusmin R, Ugochukwu UC, Mukhopadhyay R, Manjaiah KM. Modified clay minerals for environmental applications. In: Mercurio M, Sarkar B, Langella A, editors. Modified clay minerals for environmental applications. Modified clay and zeolite nanocomposite materials: environmental and pharmaceutical applications. Elsevier; 2019. p. 113–27.

[CR36] Schroder HF, Jose H, Gebhardt W, Moreira R, Pinnekamp J (2010) Biological wastewater treatment followed by physicochemical treatment for the removal of fluorinated surfactants. Water Sci Technol 61:3208–3215 20555218 10.2166/wst.2010.917

[CR37] Sorengård M, Ostblom E, Kohler S, Ahrens L (2020) Adsorption behavior of per-and polyfluoralkyl substances (PFASs) to 44 inorganic and organic sorbents and use of dyes as proxies for PFAS sorption. J Environ Chem Eng 8:103744

[CR38] Sunderland EM, Hu XC, Dassuncao C, Tokranov AK, Wagner CC, Allen JG (2019) A review of the pathways of human exposure to poly-and perfluoroalkyl substances (PFASs) and present understanding of health effects. J Exp Sci Environ Epidemiol 29:131–147 10.1038/s41370-018-0094-1 PMC6380916 30470793

[CR39] U.S. Geological Survey (n.d.) pH and water – Water Science School. Retrieved September 2, 2025, from https://www.usgs.gov/water-science-school/science/ph-and-water

[CR40] U.S. Environmental Protection Agency (2025) Drinking water regulations and contaminants. https://www.epa.gov/sdwa/drinking-water-regulations-and-contaminants

[CR41] Wang F, Liu C, Shih K (2012) Adsorption behavior of perfluorooctanesulfonate (PFOS) and perfluorooctanoate (PFOA) on boehmite. Chemosphere 89:1009–1014 22897837 10.1016/j.chemosphere.2012.06.071

[CR42] Wang Z, Cousins IT, Scheringer M, Hungerbuehler K (2015) Hazard assessment of fluorinated alternatives to long-chain perfluoroalkyl acids (PFAAs) and their precursors: status quo, ongoing challenges and possible solutions. Environ Int 75:172–179 25461427 10.1016/j.envint.2014.11.013

[CR43] Wang M, Orr AA, Jakubowski JM, Bird KE, Casey CM, Hearon SE, Tamamis P, Phillips TD (2021) Enhanced adsorption of per- and polyfluoroalkyl substances (PFAS) by edible, nutrient-amended montmorillonite clays. Water Res 188:116534. 10.1016/j.watres.2020.116534 33125992 10.1016/j.watres.2020.116534 PMC7725962

[CR44] World Health Organization (2022) Guidelines for drinking-water quality: fourth edition incorporating the first and second addenda. https://www.who.int/publications/i/item/9789240045064 35417116

[CR45] Xenophontos X, Oladele JO, Wang M, Lilly K, Martinez L, Phillips TD, Tamamis P (2025) Caffeine, riboflavin and curcumin amended clays for PFAS binding. Comput Chem Eng 201:109215 40852629 10.1016/j.compchemeng.2025.109215 PMC12369673

[CR46] Yan F, Chu YY, Zhang K, Zhang FF, Bhandari N, Ruan GD, Dai ZY, Liu Y, Zhang Z, Kan AT (2015) Determination of adsorption isotherm parameters with correlated errors by measurement error models. Chem Eng J 281:921–930

[CR47] Zhang W, Liang Y (2021) Interactions between *Lemna minor* and PFAS intermediates: perfluorooctanesulfonamide and 6:2 fluorotelomer sulfonate. Chemosphere 276:130165. 10.1016/j.chemosphere.2021.130165 33714153 10.1016/j.chemosphere.2021.130165

[CR48] Zhang X, Lohmann R, Sunderland EM (2019) Poly-and perfluoroalkyl substances in seawater and plankton from the northwestern Atlantic margin. Environ Sci Technol 53:12348–12356 31565932 10.1021/acs.est.9b03230 PMC6992416

[CR49] Zhu Y, Ji H, He K, Blaney L, Xu T, Zhao D (2022) Photocatalytic degradation of GenX in water using a new adsorptive photocatalyst. Water Res 220:118650. 10.1016/j.watres.2022.118650 35640506 10.1016/j.watres.2022.118650

