# Supplementary material for: Adsorption and detoxification of poly- and perfluoroalkyl substances (PFAS) with hydrophobic bentonite clays
Source: Environ Sci Pollut Res Int. 2026 Feb 28;33(9):4012–27. doi: 10.1007/s11356-026-37545-x (PMC13043569; doi:10.1007/s11356-026-37545-x)
Supplement: Supplementary file 1 — (DOCX 18.2 KB) [file 11356_2026_37545_MOESM1_ESM.docx]

**Supplementary material**

Table S1. Organoclay Specifications

|  | EXPANDA-TROL | AGRI-TROL B | AGRI-TROL M | OT2 |
| --- | --- | --- | --- | --- |
| Appearance | Gray to tan | Gray to tan | Gray to tan | Gray to tan |
| Bulk Density, lb/ft^3^ | 57 | 57 | 57 | 57 |
| LOI % | 34-40 | 34-40 | 34-40 | 34-40 |
| Screen analysis  % > 200 mesh | 84% Min | 84% Min | 84% Min | 84% Min |
| Specific Gravity | 1.7 | 1.7 | 1.7 | 1.7 |

*(Data collected from product information sheets)*

Table S2. Hydrophobicity of the organoclays.

| Organoclays | Hydrophobicity |
| --- | --- |
| ENT | 0.74 |
| ATB | 0.68 |
| ATM | 0.74 |
| OT2 | 1.28 |
| SB | 0.27 |

ENT: ENVIRO-TROL; ATB: AGRI-TROL B; ATM: AGRI-TROL M; OT2: ORGANO-TROL; SB: sodium bentonite clay.
